# Recipient bed perfusion as a predictor for postoperative complications in irradiated patients with microvascular free tissue transfer of the head and neck area: a clinical analysis of 191 microvascular free flaps

**DOI:** 10.1007/s10006-022-01070-1

**Published:** 2022-05-12

**Authors:** Yannick Foerster, Laura Baumann, Ino Kafantari, Manuel Olmos, Falk Wehrhan, Marco R Kesting, Raimund HM Preidl

**Affiliations:** Department of Oral and Maxillofacial Surgery, University Hospital Erlangen, University of Erlangen-Nuremberg, Glückstraße 11, 91054 Erlangen, Germany

**Keywords:** Microvascular free flap, Oxygen-to-see, Recipient bed, Radiotherapy, Monitoring, Complications

## Abstract

**Purpose:**

Despite microvascular free tissue transfer being the mainstay of care in the reconstruction of larger maxillofacial defects, a significant number of patients experience postoperative complications due to impaired blood supply of the flap. In this context, the early influence of recipient bed perfusion remains unclear, but there is evidence that it is associated with free flap viability immediately after surgery.

**Methods:**

We analyzed flap and recipient bed perfusion within the first 2 weeks after surgery by using the oxygen-to-see device. One hundred ninety-one patients who underwent free flap surgery in our department were included.

**Results:**

Flow parameters were higher and postoperative complications were less frequent in radial forearm free flaps compared to any other type of flap. Flow parameters of the recipient bed were higher than transferred tissue at all times, implicating flap autonomization is not completed within 2 weeks. Previous radiotherapy significantly decreased flow parameters of the recipient bed but not of the flaps. Furthermore, irradiated patients with postoperative complications were found to have reduced flow parameters of their recipient bed compared to non-irradiated patients with postoperative complications.

**Conclusion:**

We conclude that monitoring of recipient bed perfusion is useful for detecting flap compromise of irradiated patients in the early postoperative period.

**Supplementary Information:**

The online version contains supplementary material available at 10.1007/s10006-022-01070-1.

## Introduction

Microvascular free tissue transfer is a reliable and well-established method for the reconstruction of large composite defects in the head and neck area. Various types of free flaps have been developed and further modified in order to restore soft tissue as well as bony defects in the head and neck area [1]. This can lead to both an adequate functional and aesthetic outcome. Meeting almost any requirement, more than 20 different types of microvascular free flaps for head and neck reconstruction are available today [2, 3]. Since its introduction in 1978, the radial forearm free flap (RFFF) has increased enormously in popularity due to its relative ease of harvest, reliable anatomy, pliable tissue, and the possibility of a multi-team approach leading to decreased operation time [4, 5]. Besides the most commonly used RFFF, the free latissimus dorsi flap (FLDF) and the scapular/parascapular free flap (SPFF) are two popular options for the reconstruction of head and neck defects. Both can be harvested alone or in combination based on a single vascular pedicle which allows the resurfacing of large composite defects [6]. In contrast to the fasciocutaneous RFFF and musculocutaneous FLDF, the osteomyocutaneous SPFF is also suitable for the reconstruction of defects involving both soft and bony tissues [7].

Even though the success rate of microvascular free tissue transfer exceeds 90% [8], postoperative complications such as impaired venous or arterial flow, wound infection, or hematoma may prove devastating for the patient [9]. To avoid potential flap failure, postoperative complications must be identified early and addressed sufficiently. Monitoring of free flap perfusion with a laser Doppler and tissue oxygen measurement are reliable methods for predicting ischemia in microvascular free flaps of the head and neck [10, 11]. Albeit the early success of microvascular free tissue transfer is based on the patency of the vascular pedicle rather than on random blood supply from the surrounding recipient bed, neovascularization into the graft is indispensable to maintain flap viability over time [12]. During this autonomization process, the free flap becomes independent from the vascular pedicle. However, there is no consent about when free flap autonomization is completed [13–19]. As neovascularization is sprawling from the surrounding recipient bed into the flap, a good condition of the recipient bed is a prerequisite for successful free flap autonomization, and there is evidence that recipient bed vascularity may contribute to survival of ischemic microvascular free flaps in rats immediately after surgery [20]. However, the effects of recipient bed perfusion on postoperative complications remain unclear. In addition, the impact of previous radiotherapy on the recipient bed of the free flap is poorly understood in the context of flap autonomization, but it is well known that previous radiotherapy is associated with a higher risk of flap failure, wound-healing disorders, and complications due to endothelial alterations of the local vessels leading to an impaired blood supply [21–28]. Therefore, the aim of the present study was to investigate differences between free flap and recipient bed perfusion with regard to postoperative complications within the first 2 weeks after microvascular free tissue transfer. The effects of a previous radiotherapy as well as differences between the various flap entities were also evaluated. We also investigated whether there was any sign of free flap autonomization during the first 2 weeks after surgery by evaluating free flap and recipient bed perfusion parameters, respectively.

## Material and methods

This study included 191 patients who have undergone microvascular free tissue transfer in the Department of Oral and Maxillofacial Surgery at the University Hospital Erlangen, Germany, between October 2013 and July 2015. The patient population is described in Table 1. The study protocol was approved by the local ethical committee at Friedrich-Alexander-University of Erlangen-Nuremberg, Germany (No. 83_13B).

**Table 1:** Baseline patient data. Total cases, mean patient age ± standard deviation (SD), gender distribution, previous radiotherapy (irradiation), indications for microvascular tissue transfer including primary tumor, recurrent tumor, osteoradionecrosis of the jaw (ORNJ), wound healing deficit and metastasis

Free flap entity	Cases [%]	Age mean ± SD [y]	Gender		Indication for surgery					Irradiated [%]
			Male [%]	Female [%]	Primary tumor [%]	Recurrent tumor [%]	ORNJ [%]	Wound healing deficit [%]	Metastasis [%]	
Total	191/191 [100]	63.11 ± 12.66	142/191 [74.3]	49/191 [25.7]	117/191 [61.3]	31/191 [16.2]	23/191 [12]	19/191 [10]	1/191 [0.5]	55/191 [28.8]
Radial forearm free flap (RFFF)	103/191 [53.9]	63.25 ± 11.98	72/103 [69.9]	31/103 [30.1]	73/103 [70.9]	17/103 [16.5]	7/103 [6.8]	6/103 [5.8]	0/103 [0]	20/103 [19.4]
Free fibula flap (FFF)	10/191 [5.2]	51.8 ± 16.12	6/10 [60]	4/10 [40]	2/10 [20]	1/10 [10]	2/10 [20]	5/10 [50]	0/10 [0]	4/10 [40]
Scapula/parascapular free flap (SPFF)	25/191 [13.1]	62.48 ± 13.72	20/25 [80]	5/25 [20]	12/25 [48]	4/25 [16]	6/25 [24]	3/25 [12]	0/25 [0]	14/25 [56]
Free latissimus dorsi flap (FLDF)	42/191 [22]	63.74 ± 11.86	35/42 [83.3]	7/42 [16.7]	24/42 [57.1]	7/42 [16.7]	7/42 [16.7]	3/42 [7.1]	1/42 [2.4]	14/42 [33.3]
Lateral arm flap (LAF)	8/191 [4.2]	70.88 ± 11.03	6/8 [75]	2/8 [25]	4/8 [50]	2/8 [25]	1/8 [12.5]	1/8 [12.5]	0/8 [0]	2/8 [25]
Anterolateral thigh free flap (ALT)	3/191 [1.6]	71.67 ± 12.86	3/3 [100]	0/3 [0]	2/3 [66.7]	0/3 [0]	0/3 [0]	1/3 [33.3]	0/3 [0]	1/3 [33.3]

**Fig. 1:**
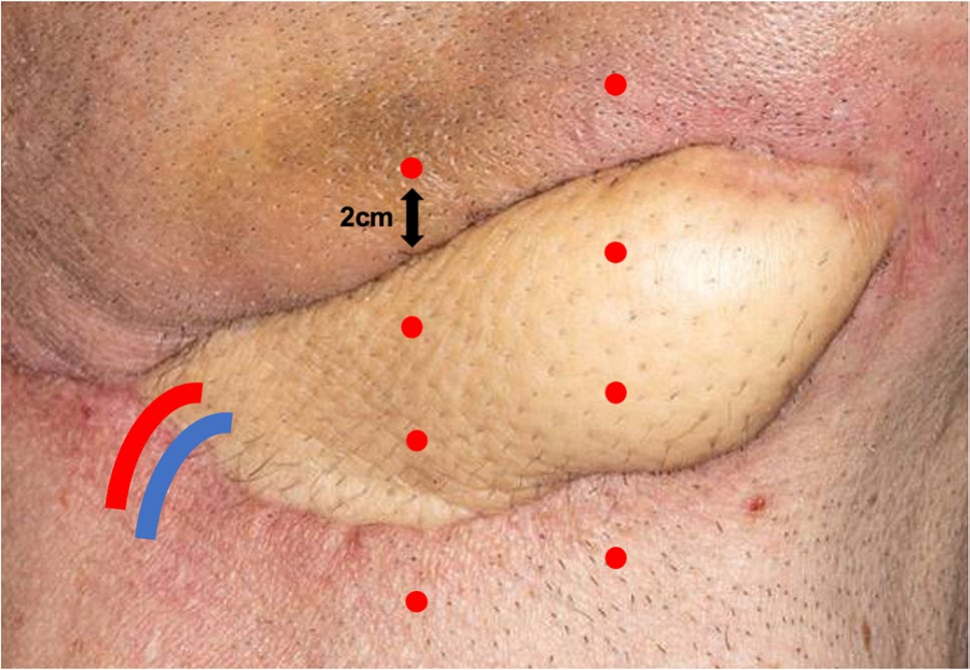
Measuring points. Measuring points within and outside the skin island of a microvascular free flap. A total of eight different points were measured (four points within and four points outside the skin island). Measuring points outside the skin island were located 2 cm from the outer margin in the recipient bed. Axial pedicle is indicated on the left

Four different points on the skin island of the free flap were measured. Additionally, in a subgroup of 101 free flaps, four standardized points in the recipient bed lying 2 cm distant to the outer margin of the free flap were included (Fig. 1). Standardized measurements were performed with oxygen-to-see (O2C, Lea Medizintechnik, Gießen, Germany) over an interval of 15 s on each of the selected points at postoperative days 1, 2, 3, 5, 7, and 14 after the patient was at rest and positioned horizontally for at least 10 min. Flap perfusion was quantified by white light spectroscopy (venous partial oxygen saturation of hemoglobin (SpO_2_), relative amount of hemoglobin (Hb)) and laser Doppler (blood flow (AU = arbitrary units) and flow velocity (AU)) at 2 mm depth simultaneously. Measuring of perfusion parameters by white light spectroscopy is based on a Doppler shift caused by moving red blood cells which broaden the spectrum of the reflected light. SpO_2_ and Hb are determined using tissue spectrometry. The light emitted into the tissue is absorbed or scattered depending on the Hb and SpO_2_ [10].

### Statistical analysis

Comparison between different groups was performed using cross-supplemental tables (chi2-test), unpaired t-test, or one-way ANOVA, including the Bonferroni post hoc test (SPSS Version 27.0, SPSS Inc., Chicago, IL, USA). Data is expressed in mean ± standard deviation (SD) and/or in combination with the confidence interval (CI) at a confidence level of 95%. No imputations of missing values were done. A p value less than 0.05 was considered statistically significant (*p* < 0.05*; *p* < 0.01**; *p* < 0.001***).

## Results

### Radial forearm free flaps show higher blood flow and velocity parameters than scapula/parascapular and free latissimus dorsi flaps

**Fig. 2:**
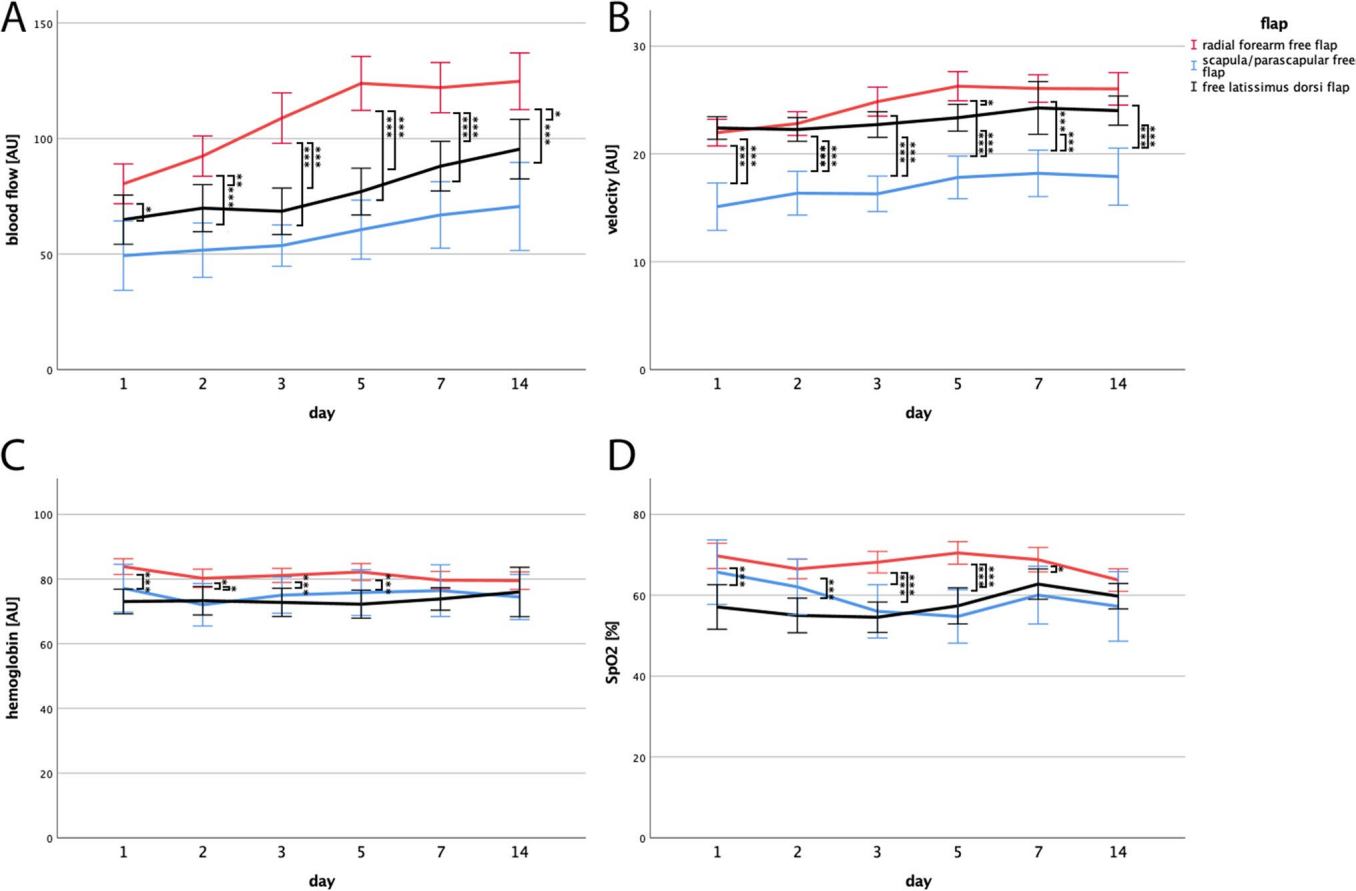
Blood flow, velocity, hemoglobin and oxygen saturation curves of RFFF, SPFF, and FLDF. Changes of mean blood flow (**A**), velocity (**B**), relative amount of hemoglobin (**C**), and venous partial oxygen saturation of hemoglobin (**D**) during the first two postoperative weeks after receiving microvascular tissue transfer with radial forearm free flap (RFFF), scapula/parascapular free flap (SPFF), or free latissimus dorsi flap (FLDF). RFFF exhibited higher blood flow values compared to SPFF and FLDF at almost all time points. Regarding velocity, RFFF and FLDF only varied on day 5 but both turned out to have significantly higher values compared to SPFF at all time points. Compared to FLDF, RFFF revealed a higher oxygen saturation and amount of hemoglobin during the early 7 days after surgery (*p* < 0.05*; *p* < 0.01**; *p* < 0.001***) (mean + 95%CI)

Blood flow and velocity parameters of RFFF and FLDF significantly increased during the postoperative time period in linear regression analysis, whereas SPFF only revealed a statistically significant increase in blood flow (RFFF: blood flow and velocity: *p* < 0.001; FLDF: blood flow: *p* < 0.001, velocity: *p* = 0.035; SPFF: blood flow: *p* = 0.008). Also, RFFF exhibited a significantly higher blood flow compared to SPFF (day 1: *p* = 0.003; days 2–14: *p* < 0.001) and FLDF (day 2: *p* = 0.007; days 3–7: *p* < 0.001; day 14: *p* = 0.014) at almost all investigated time points (Fig. 2A, Table S1). Only on the first postoperative day, blood flow parameters of RFFF and FLDF did not differ significantly. Although mean blood flow levels of FLDF were higher compared to SPFF, this difference was not statistically significant. In contrast, velocity parameters of both RFFF and FLDF were significantly higher compared to SPFF at all investigated time points (*p* < 0.001 all), while velocity parameters of RFFF and FLDF only varied on the fifth postoperative day (Fig. 2B, Table S1) (*p* = 0.024). Regarding the relative amount of Hb and its SpO_2_, we could not observe any increase during the investigated postoperative period. Moreover, the parameters between the three flap entities only varied during the first week after surgery. The Hb of RFFF compared to FLDF turned out to be significantly higher on the first 5 days after surgery (days 1, 3, 5: *p* < 0.001; day 2: *p* = 0.03). Additionally, RFFF exhibited a significantly higher Hb than SPFF on the second postoperative day (*p* = 0.042). Hb of FLDF and SPFF did not vary at any time (Fig. 2C, Table S1). Focusing on SpO_2_, similar results can be found. Particularly, RFFF revealed significantly higher SpO_2_ parameters than FLDF on the first five days after surgery (*p* < 0.001 all) and in comparison with SPFF, SpO_2_ of RFFF was significantly higher on postoperative days 3–7 (days 3, 5: *p* < 0.001; day 7: *p* = 0.029). SPFF and FLDF did not differ in terms of SpO2 (Fig. 2D, Tab. S1). Due to the low number of patients who received head and neck reconstruction with flaps that were not RFFF, FLDF, and SPFF, we focused on those entities for this part of the study.

### Radial forearm free flap is associated with less postoperative complications compared to other investigated flap entities

Compared to the other investigated flap entities, RFFF showed less postoperative complications (*p* = 0.022) (Table 2). A total of 20.4% (21/103) of patients who received head and neck reconstruction with RFFF suffered from postoperative complications, whereas, on average, 35.2% (31/88) of patients with a flap entity other than RFFF experienced postoperative complications. The highest rate of postoperative complications was in patients with a lateral arm flap (5/8, 62.5%) or an anterolateral thigh flap (2/3, 66.7%). Most common postoperative complications in RFFF were hematoma or wound healing deficits, each affecting 4.9% (5/103) of patients with RFFF. Overall, 14.6% (15/103) of RFFF and 22.3% (20/88) of the other free flap entities had to be surgically revised within the first 2 weeks after the initial microvascular free tissue transfer. However, this difference was not statistically significant. Only one free flap (FLDF) was lost during the investigated period. Furthermore, previous radiotherapy in the head and neck area had an impact on the frequency of postoperative complications. In particular, postoperative complications occurred in 36.4% (20/55) of irradiated and only in 23.5% (32/136) of non-irradiated patients (*p* = 0.071). This difference was found independent from type of free flap. A more detailed overview of the respective postoperative complications with regard to the different free flap entities is given in Table 2.

**Table 2:** Postoperative complications dependent on free flap entity

Free flap entity	Postoperative complication								
	Total [%]	Hematoma [%]	Infection [%]	Flap failure [%]	Necrosis [%]	Revision artery [%]	Revision vein [%]	Prolonged wound healing [%]	Surgical revision overall [%]
Total	52/191 [27.2]	13/191 [6.8]	4/191 [2.1]	1/191 [0.5]	6/191 [3.1]	8/191 [4.2]	7/191 [3.7]	13/191 [6.8]	35/191 [18.3]
Radial forearm free flap (RFFF)	21/103 [20.4]	5/103 [4.9]	1/103 [1]	0/103 [0]	4/103 [3.9]	4/103 [3.9]	2/103 [1.9]	5/103 [4.9]	15/103 [14.6]
Free fibula flap (FFF)	3/10 [30]	0/10 [0]	1/10 [10]	0/10 [0]	0/10 [0]	0/10 [0]	0/10 [0]	2/10 [20]	0/10 [0]
Scapula/parascapular free flap (SPFF)	9/25 [36]	3/25 [12]	1/25 [4]	0/25 [0]	1/25 [4]	0/25 [0]	1/25 [4]	3/25 [12]	5/25 [20]
Free latissimus dorsi flap (FLDF)	12/42 [28.6]	5/42 [11.9]	0/42 [0]	1/42 [2.4]	0/42 [0]	0/42 [0]	4/42 [9.5]	2/42 [4.8]	10/42 [23.8]
Lateral arm flap (LAF)	5/8 [62.5]	0/8 [0]	1/8 [12.5]	0/8 [0]	0/8 [0]	3/8 [37.5]	0/8 [0]	1/8 [12.5]	3/8 [37.5]
Anterolateral thigh free flap (ALT)	2/3 [66.7]	0/3 [0]	0/3 [0]	0/3 [0]	1/3 [33.3]	1/3 [33.3]	0/3 [0]	0/3 [0]	2/3 [66.7]

### Previous radiotherapy decreases blood flow of the recipient bed

**Fig. 3:**
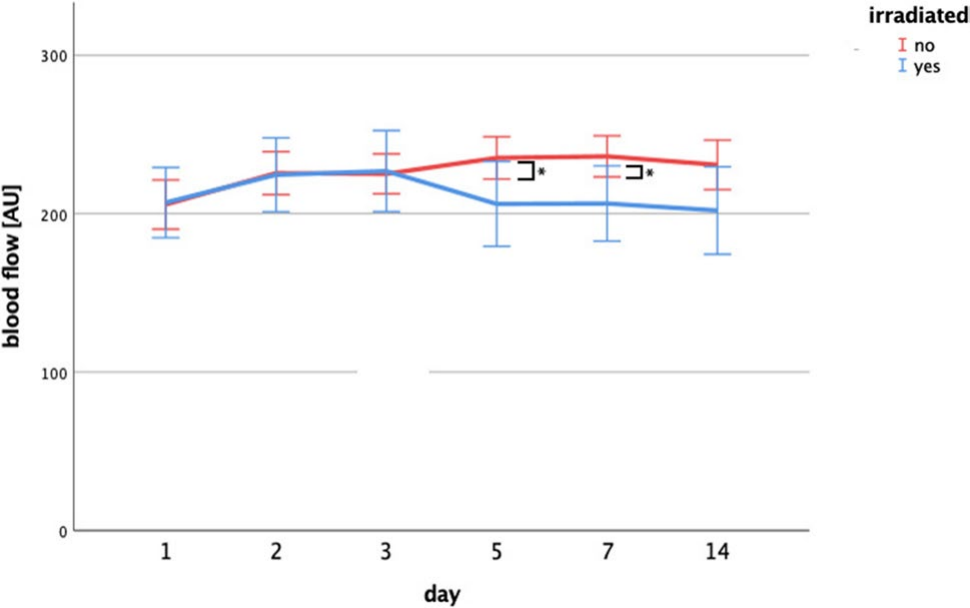
Blood flow curve of the recipient bed with regard to previous radiotherapy. Changes of mean blood flow in the recipient bed dependent on previous radiotherapy. Mean blood flow of the irradiated recipient bed was significantly reduced compared to the non-irradiated recipient bed on the fifth (*p* = 0.031*) and seventh (*p* = 0.03*) postoperative day (mean + 95%CI)

The investigated parameters were also evaluated with respect to previous radiotherapy. All patients were subdivided into two groups depending on whether or not they had received radiation of the head and neck area prior to surgery. Focusing on the recipient bed, patients who had received radiotherapy in their past exhibited significantly reduced blood flow on the fifth and seventh postoperative day compared to non-irradiated patients (day 5: *p* = 0.031; day 7: *p* = 0.03) (Fig. 3; Table S2). This difference was not seen during the first three postoperative days. No other parameters of the recipient bed, such as velocity, Hb or SpO_2_ were altered by preoperative radiotherapy (Table S2). No increase in blood flow was found in the irradiated or the non-irradiated recipient beds.

On the other hand, mean blood flow parameters of the free tissue transfer increased during the postoperative period in linear regression analysis independent from previous radiotherapy (*p* < 0.001 both) (Table S2). There is no difference in the flap’s mean blood flow when comparing irradiated and non-irradiated patients (Table S2). According to the recipient bed, we could not observe any other differences comprising velocity, Hb or SpO_2_ (Table S2). Overall, no difference between the various free flap entities could be found.

### Recipient bed shows higher blood flow, velocity, relative amount of hemoglobin and partial oxygen saturation of hemoglobin compared to the free flap within the first two weeks after surgery

**Fig. 4:**
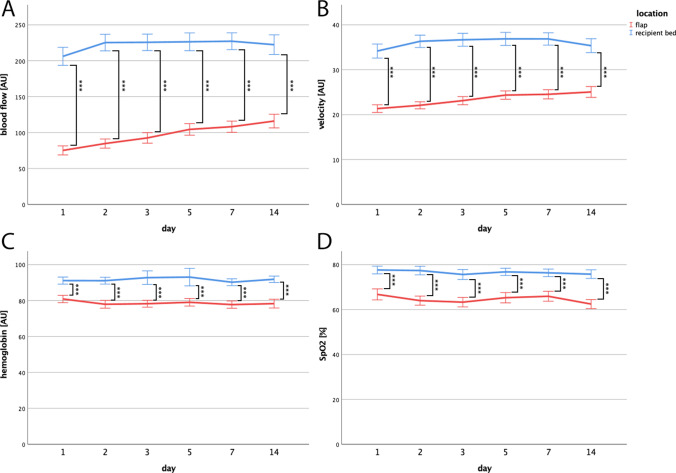
Blood flow, velocity, hemoglobin and oxygen saturation curves of the recipient bed and the free flap. Changes of mean blood flow (**A**), velocity (**B**), relative amount of hemoglobin (**C**), and venous partial oxygen saturation of hemoglobin (**D**) in the recipient bed and the free flap. The recipient bed revealed higher parameters compared to the free flaps at all investigated time points (*p* < 0.001***) (mean + 95%CI)

Mean blood flow, velocity, Hb, and SpO_2_ of the surrounding recipient bed were significantly higher compared to the flap, independent from free flap type (all *p* < 0.001) (Fig. 4, Table S3). This includes all investigated time points within 2 weeks after the patients received a microvascular free tissue transfer. Summarizing all investigated microvascular free flaps, they showed a significant increase of blood flow and velocity parameters during the postoperative period in linear regression analysis (both *p* < 0.001). In fact, mean blood flow and velocity parameters of the flap and the recipient bed apparently approximated during the immediate postoperative period but the differences between both areas were still statistically significant 2 weeks after surgery. Neither Hb nor SpO_2_ showed a significant increase or decrease during the investigated period in linear regression analysis.

### Irradiated patients with postoperative complications show decreased blood flow of the recipient bed compared to non-irradiated patients with postoperative complications

**Fig. 5:**
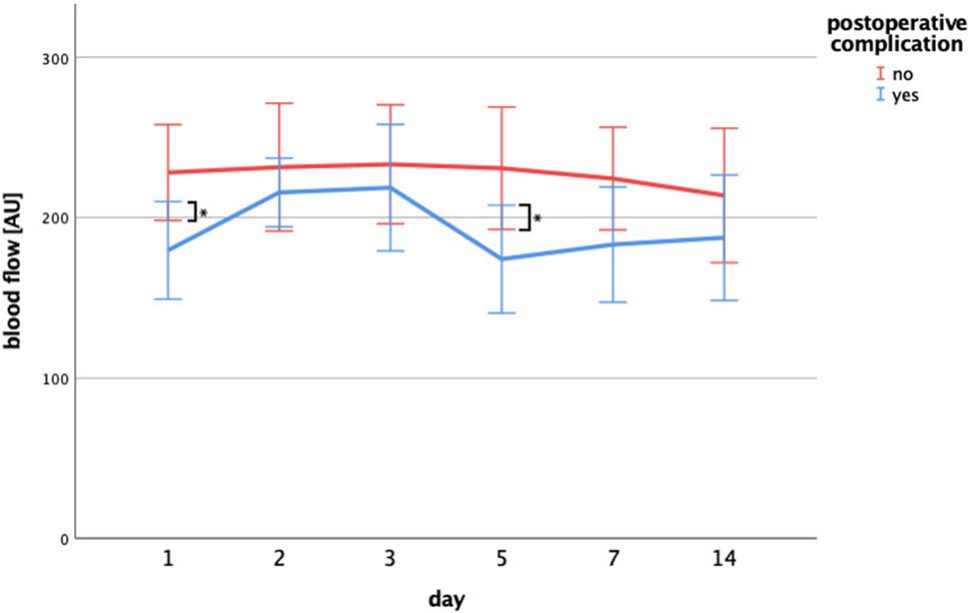
Blood flow curves of the recipient bed with regard to a previous radiotherapy and the occurrence of postoperative complications. Changes of mean blood flow in the recipient bed of patients who received previous radiotherapy. Irradiated patients who experienced a postoperative complication revealed significantly decreased mean blood flow parameters compared to patients lacking a postoperative complication (day 1: *p* = 0.021*; day 5: *p* = 0.024*) (mean + 95%CI)

In order to further evaluate the influence of previous radiotherapy on postoperative blood flow parameters and complications of the recipient bed and the flap, respectively, we subdivided the patients into the following groups: mean blood flow of the recipient bed of irradiated patients (30 patients), mean blood flow of the recipient bed of non-irradiated patients (72 patients), mean blood flow of the free flap of irradiated patients (55 patients) and mean blood flow of the free flap of non-irradiated patients (136 patients). In each group, mean blood flow parameters of patients suffering from a postoperative complication were compared with blood flow parameters of patients who did not have any postoperative complication. Focusing on the recipient bed, irradiated patients who experienced a postoperative complication revealed significantly decreased mean blood flow parameters compared to patients who did not have a postoperative complication (day 1: *p* = 0.021; day 5: *p* = 0.024) (Fig. 5, Table S4). In contrast, there was no difference between the flap’s mean blood flow parameters of patients with and without postoperative complications (Table S4). Neither irradiated nor non-irradiated recipient beds showed an increase of mean blood flow parameters during the investigated time period in linear regression analysis.

In contrast, we could not find similar results when focusing on the flap rather than the recipient bed. Particularly, we did not see any difference between blood flow parameters of the flap in either irradiated or in non-irradiated patients (Table S4). However, all flaps revealed significant increases of mean blood flow parameters in linear regression analysis (irradiated plus complication: *p* = 0.045; irradiated without complication: *p* = 0.005; non-irradiated plus complication: *p* < 0.001; non-irradiated without complication: *p* < 0.001). With regard to the other investigated parameters, including velocity, Hb, and SpO_2_, we could not find any difference at all.

## Discussion

Survival rates of microvascular free flaps range between 90 and 98% [29]. Besides relying on physical examination assessing color, turgor or capillary refill time, adjunctive techniques like laser Doppler of the vascular pedicle have proven to be a useful tool for evaluating free flap viability [30] and there is evidence that also perfusion of the surrounding recipient bed correlates with free flap survival not only in the long run, but also within the early postoperative days [20, 31]. Therefore, the present study is the first to investigate blood flow, flow velocity, hemoglobin concentration, and tissue oxygenation parameters of microvascular free flaps and the surrounding recipient bed within the first 2 weeks after surgery by using the O2C device, which delivers reliable real-time perfusion data via a non-invasive technique [10]. This includes parameters of irradiated and non-irradiated patients and both are considered with regard to the appearance of postoperative complications. We found that mean blood flow values of the recipient bed of irradiated patients were significantly lower compared to non-irradiated patients. Furthermore, it turned out that blood flow parameters of the irradiated recipient bed were significantly lower in patients who experienced early postoperative complications compared to patients who were not affected by a complication during the postoperative course. Remarkably, this difference was already present within the first postoperative day, which leads to the conclusion that sufficient recipient bed perfusion is essential for microvascular free flap viability immediately after surgery. In line with these observations, we conclude that monitoring of the recipient bed perfusion is particularly useful for the early prediction of postoperative complications in irradiated patients.

When comparing the overall blood flow and velocity parameters of the free flap and the recipient bed, we could observe an approximation of both curves, but the differences were still statistically significant at the end of the investigated period. This leads to the conclusion that there is a process of autonomization of the free flap, but it is not fully completed within the first 14 postoperative days.

Overall, a total of 18.3% (35/191) of patients required surgical revision but only one single flap failure could be observed in our study group, indicating the importance of early detection and operative intervention for flap salvage. In accordance with the current literature [32], we observed a higher number of postoperative complications in patients who had received radiotherapy before the surgery (36.4% in irradiated compared to 23.5% in non-irradiated patients).

**Fig. 6:**
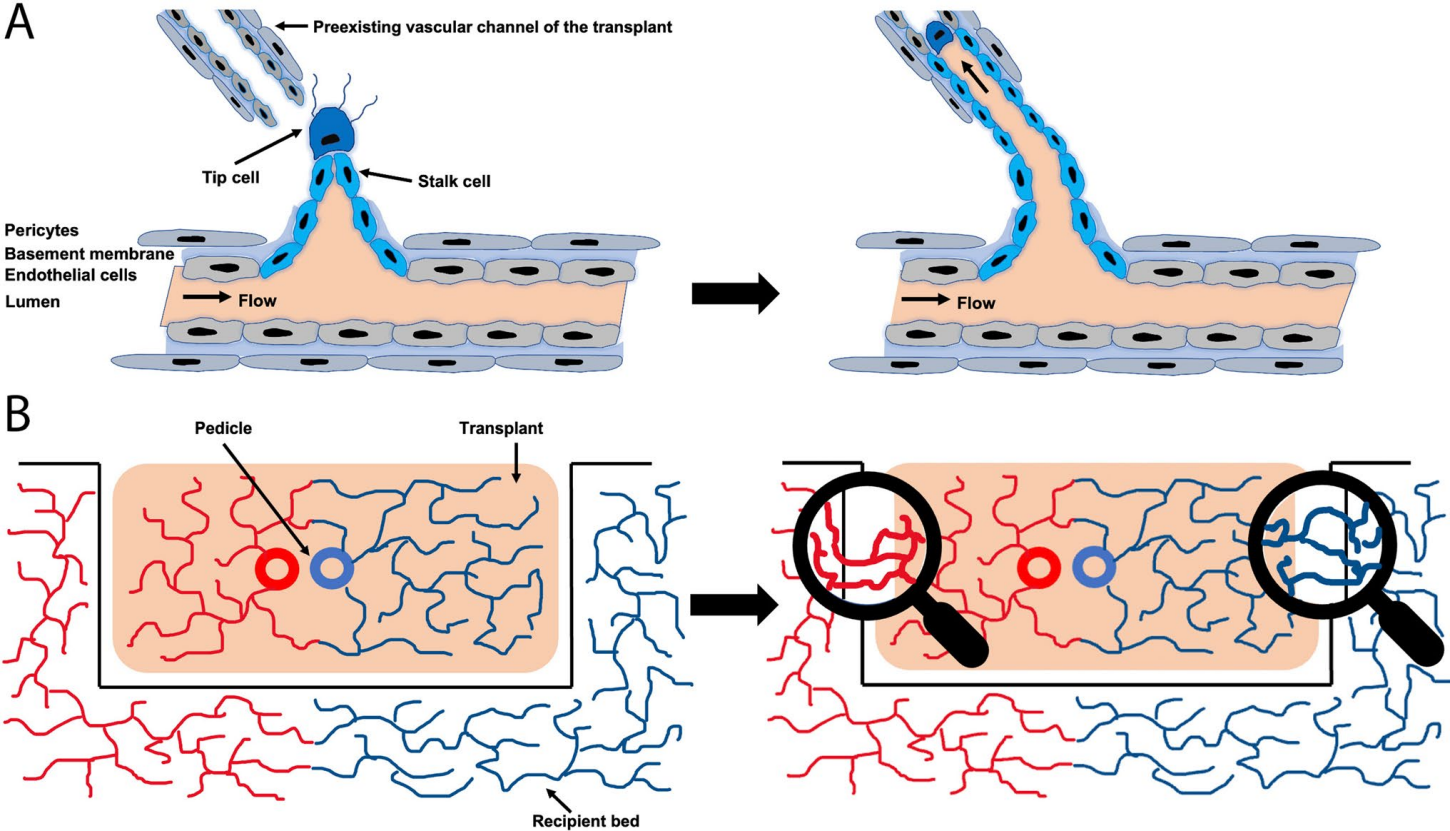
Inosculation of free flaps. During the early days after surgery, the free flap (transplant) is supplied with blood only via the vascular pedicle. Over time, endothelial cells of the recipient bed, mediated through tip-stalk cell selection and shuffling, invade in preexisting vascular channels of the flap (**A**), leading to a second vascular connection of the flap and the recipient bed independent from the pedicle (**B**)

Our data show that perfusion parameters, especially flow and velocity, significantly vary in different types of free microvascular flaps, which has to be taken into consideration during postoperative monitoring. In particular, RFFF shows higher blood flow and velocity parameters compared to SPFF and FLDF. The O2C device detects flow parameters in the capillaries at 2-mm depth [10, 33]. Therefore, varying flow parameters might be explained by substantial differences in flap volume and weight, with RFFF being smaller and lighter compared to SPFF and FLDF. Regarding the postoperative course of flow and velocity in the present study, a significant increase in blood flow during the investigating period was observed. But reaching postoperative day 5, a reduced increase or even a slight decrease with a consecutive flattening of the flow- and velocity- curves of RFFF could be observed. We hypothesize that during the first postoperative days, the reconnection of the existing capillaries known as inosculation has not yet occurred so free flaps are exclusively supplied via the flap pedicle. During this process endothelial cells of the recipient bed, mediated through tip-stalk cell selection and shuffling [34], invade in preexisting vascular channels of the flap. As a result, this process leads to further blood supply independent from the vascular pedicle (Fig. 6) and different studies suggest that accelerating the connection of those life-sustaining pipelines may help to enhance the viability of implanted tissue constructs [35, 36]. Conclusively, the flattening of the flow and velocity curve might be associated with a successfully ongoing inosculation and therefore an autonomization of free flaps with regard to the blood supply. However, we did not particularly evaluate neovascularization, so the approximation of both curves may also be a result of autoregulatory processes within the flap and the recipient bed. We could not observe similar flattening of blood flow and velocity parameters in SPFF and FLDF, indicating that inosculation and flap autonomization may be delayed compared to RFFF. In accordance with other studies [10, 14], our results show neither an increase in relative hemoglobin nor in venous partial oxygen saturation. On the contrary, all flaps even show a slight decrease of SpO_2_ from the seventh postoperative day, which we hypothesized could be due to postoperative healing processes associated with local inflammatory responses.

After reviewing the current literature, most studies show only minor differences with a slight superiority of RFFF in terms of recipient site survival or postoperative complications when comparing various free flaps for head and neck reconstruction [37–40], which is consistent with our observations. There is broad consensus that free flap viability mainly relies on the axial blood supply through the vascular pedicle on the early postoperative days [41]. In this critical time frame, obstruction of the vascular pedicle can lead to flap failure and may prove disastrous for the patient. Once the ongoing inosculation reaches a critical threshold, the flap becomes independent from the vascular pedicle. As mentioned above, we hypothesized that inosculation and autonomization of RFFF proceeds earlier in the postoperative period compared to FLDF and SPFF, which could explain the lower number of postoperative complications we have seen in RFFF compared to other free flap entities. RFFF is the most frequently used free flap entity in our department which, in fact, could also lead to a lower complication rate due to a higher expertise.

Moreover, higher mean blood flow values of the non-irradiated recipient bed compared to the irradiated recipient bed were not present until the fifth postoperative day, indicating that this could be the first sign of blood flow through the capillaries from the surrounding recipient bed into the flap and therefore the beginning of a proceeding inosculation, which is consistent with other studies, suggesting free flap autonomization between the postoperative days 5–15 [13–18]. This correlates with our previous observation that blood flow and velocity parameters decrease from postoperative day five on, which we attribute to successfully ongoing inosculation. However, there are no differences between the various free flap entities, which is contrary to our hypothesis that autonomization of RFFF may start earlier compared to FLDF and SPFF.

Previously, we concluded that impaired blood flow parameters are probably a result of endothelial alterations due to prior radiation. If our hypothesis is true, this difference should not show up in non-irradiated recipient beds and this is what we have seen in our study. However, we could only show a correlation between reduced recipient bed perfusion and the occurrence of complications in the early postoperative period. Therefore, further studies must clarify the particular function of recipient bed perfusion for free flap viability in the early days after surgery. Also the link between approximating flow curves and histological alterations should be further investigated.

## Supplementary Information

Below is the link to the electronic supplementary material.

Supplementary file1 (DOCX 26 KB)
