# Supplementary material for: Recipient bed perfusion as a predictor for postoperative complications in irradiated patients with microvascular free tissue transfer of the head and neck area: a clinical analysis of 191 microvascular free flaps
Source: Oral Maxillofac Surg. 2022 May 12;27(2):313–23. doi: 10.1007/s10006-022-01070-1 (PMC10235148; doi:10.1007/s10006-022-01070-1)
Supplement: Supplementary file 1 — Supplementary file1 (DOCX 26 KB) [file 10006_2022_1070_MOESM1_ESM.docx]

|  | radial forearm free flap (RFFF) | | | | | | | | scapula/parascapular free flap (SPFF) | | | | | | | | free latissimus dorsi flap (FLDF) | | | | | | | |
| --- | --- | --- | --- | --- | --- | --- | --- | --- | --- | --- | --- | --- | --- | --- | --- | --- | --- | --- | --- | --- | --- | --- | --- | --- |
| day | blood flow [AU] | | velocity [AU] | | hemoglobin [AU] | | SpO2 [%] | | blood flow [AU] | | velocity [AU] | | hemoglobin [AU] | | SpO2 [%] | | blood flow [AU] | | velocity [AU] | | hemoglobin [AU] | | SpO2 [%] | |
|  | mean | SD | mean | SD | mean | SD | mean | SD | mean | SD | mean | SD | mean | SD | mean | SD | mean | SD | mean | SD | mean | SD | mean | SD |
| 1 | 80,4 | 44,1 | 22,0 | 6,3 | 83,9 | 12,5 | 69,7 | 15,7 | 49,4 | 34,7 | 15,1 | 5,1 | 77,2 | 17,1 | 65,7 | 17,5 | 64,9 | 33,7 | 22,4 | 3,3 | 73,1 | 12,1 | 57,1 | 17,5 |
| 2 | 92,4 | 44,3 | 22,8 | 5,6 | 80,3 | 14,4 | 66,5 | 12,2 | 51,7 | 27,9 | 16,4 | 4,8 | 72,1 | 15,6 | 62,1 | 15,9 | 69,9 | 32,7 | 22,3 | 3,5 | 73,3 | 14,1 | 55,0 | 13,7 |
| 3 | 108,9 | 54,1 | 24,9 | 6,7 | 81,1 | 10,8 | 68,2 | 13,1 | 53,7 | 21,7 | 16,3 | 4,0 | 75,0 | 13,5 | 56,0 | 14,9 | 68,6 | 32,2 | 22,7 | 3,8 | 72,8 | 14,0 | 54,6 | 12,1 |
| 5 | 123,9 | 57,9 | 26,3 | 6,7 | 82,2 | 13,0 | 70,5 | 13,6 | 60,6 | 31,0 | 17,8 | 4,8 | 75,8 | 17,1 | 54,8 | 14,5 | 77,1 | 32,0 | 23,4 | 3,9 | 72,3 | 13,7 | 57,4 | 14,1 |
| 7 | 122,1 | 52,8 | 26,1 | 6,2 | 79,7 | 13,2 | 68,8 | 14,5 | 66,9 | 34,0 | 18,2 | 5,1 | 76,4 | 18,9 | 60,0 | 16,1 | 88,1 | 34,0 | 24,3 | 7,7 | 73,9 | 11,0 | 62,8 | 11,9 |
| 14 | 124,8 | 57,9 | 26,0 | 7,1 | 79,5 | 12,8 | 63,8 | 13,0 | 70,6 | 44,1 | 17,9 | 6,1 | 74,5 | 16,1 | 57,3 | 18,9 | 95,4 | 38,1 | 24,0 | 4,0 | 76,0 | 22,6 | 59,8 | 9,3 |

**Table S1**

|  | recipient bed irradiated | | | | | | | | recipient bed non-irradiated | | | | | | | | flap irradiated | | | | | | | | flap non-irradiated | | | | | | | |
| --- | --- | --- | --- | --- | --- | --- | --- | --- | --- | --- | --- | --- | --- | --- | --- | --- | --- | --- | --- | --- | --- | --- | --- | --- | --- | --- | --- | --- | --- | --- | --- | --- |
| day | blood flow [AU] | | velocity [AU] | | hemoglobin [AU] | | SpO2 [%] | | blood flow [AU] | | velocity [AU] | | hemoglobin [AU] | | SpO2 [%] | | blood flow [AU] | | velocity [AU] | | hemoglobin [AU] | | SpO2 [%] | | blood flow [AU] | | velocity [AU] | | hemoglobin [AU] | | SpO2 [%] | |
|  | mean | SD | mean | SD | mean | SD | mean | SD | mean | SD | mean | SD | mean | SD | mean | SD | mean | SD | mean | SD | mean | SD | mean | SD | mean | SD | mean | SD | mean | SD | mean | SD |
| 1 | 207,0 | 59,3 | 35,0 | 7,2 | 89,0 | 9,4 | 76,2 | 8,7 | 205,8 | 65,6 | 33,8 | 8,3 | 92,0 | 10,1 | 78,2 | 8,7 | 71,9 | 38,1 | 20,8 | 4,7 | 78,2 | 13,9 | 65,3 | 16,0 | 76,5 | 45,5 | 21,5 | 6,4 | 81,9 | 14,3 | 67,3 | 17,0 |
| 2 | 224,6 | 62,5 | 36,8 | 7,3 | 92,1 | 8,3 | 77,3 | 10,0 | 225,6 | 57,7 | 36,1 | 6,9 | 90,6 | 10,2 | 77,4 | 9,7 | 83,5 | 41,0 | 22,0 | 5,1 | 76,4 | 12,7 | 66,2 | 12,3 | 85,3 | 45,6 | 22,1 | 5,6 | 78,6 | 15,9 | 63,1 | 14,6 |
| 3 | 226,9 | 68,6 | 38,1 | 8,9 | 91,5 | 8,6 | 76,1 | 12,6 | 225,2 | 53,2 | 36,1 | 6,4 | 93,3 | 22,1 | 75,4 | 10,6 | 90,2 | 49,2 | 23,0 | 6,7 | 77,0 | 11,7 | 65,7 | 13,7 | 93,6 | 51,0 | 23,2 | 6,1 | 78,8 | 13,8 | 62,4 | 14,6 |
| 5 | 206,2 | 71,7 | 34,9 | 7,7 | 99,5 | 42,3 | 77,3 | 9,1 | 235,2 | 55,6 | 37,7 | 6,9 | 90,3 | 8,6 | 76,6 | 7,6 | 102,2 | 55,7 | 24,1 | 6,4 | 78,5 | 15,5 | 66,7 | 15,2 | 105,4 | 55,3 | 24,4 | 6,4 | 79,3 | 14,1 | 64,7 | 15,5 |
| 7 | 206,5 | 63,6 | 35,0 | 7,1 | 89,0 | 9,9 | 75,8 | 9,2 | 236,2 | 54,1 | 37,7 | 6,7 | 90,7 | 9,4 | 76,6 | 8,0 | 97,5 | 46,1 | 24,6 | 8,3 | 77,8 | 14,7 | 64,9 | 16,9 | 112,9 | 55,1 | 24,5 | 6,2 | 77,8 | 13,4 | 66,4 | 13,9 |
| 14 | 202,0 | 69,8 | 33,7 | 7,1 | 91,5 | 9,7 | 75,5 | 8,8 | 230,9 | 63,0 | 36,0 | 7,6 | 92,0 | 8,1 | 75,9 | 9,5 | 106,0 | 52,2 | 25,2 | 9,6 | 75,4 | 14,2 | 61,6 | 14,1 | 119,9 | 65,4 | 25,0 | 7,2 | 79,4 | 16,4 | 62,8 | 12,7 |

**Table S2**

|  | recipient bed | | | | | | | | flap | | | | | | | |
| --- | --- | --- | --- | --- | --- | --- | --- | --- | --- | --- | --- | --- | --- | --- | --- | --- |
| day | blood flow [AU] | | velocity [AU] | | hemoglobin [AU] | | SpO2 [%] | | blood flow [AU] | | velocity [AU] | | hemoglobin [AU] | | SpO2 [%] | |
|  | mean | SD | mean | SD | mean | SD | mean | SD | mean | SD | mean | SD | mean | SD | mean | SD |
| 1 | 206,2 | 63,5 | 34,2 | 7,9 | 91,1 | 10,0 | 77,6 | 8,7 | 75,2 | 43,5 | 21,3 | 5,9 | 80,9 | 14,2 | 66,8 | 16,7 |
| 2 | 225,3 | 58,9 | 36,3 | 7,0 | 91,1 | 9,6 | 77,4 | 9,7 | 84,8 | 44,2 | 22,1 | 5,5 | 78,0 | 15,0 | 64,0 | 14,0 |
| 3 | 225,7 | 57,9 | 36,7 | 7,3 | 92,8 | 19,0 | 75,6 | 11,2 | 92,6 | 50,4 | 23,1 | 6,3 | 78,3 | 13,2 | 63,3 | 14,4 |
| 5 | 226,4 | 62,0 | 36,9 | 7,3 | 93,1 | 24,5 | 76,8 | 8,1 | 104,5 | 55,3 | 24,4 | 6,4 | 79,1 | 14,5 | 65,3 | 15,4 |
| 7 | 227,2 | 58,5 | 36,9 | 6,9 | 90,2 | 9,5 | 76,4 | 8,4 | 108,2 | 52,9 | 24,5 | 6,9 | 77,8 | 13,8 | 65,9 | 14,9 |
| 14 | 222,4 | 66,0 | 35,4 | 7,5 | 91,9 | 8,5 | 75,8 | 9,2 | 116,1 | 62,2 | 25,1 | 7,9 | 78,3 | 15,9 | 62,5 | 13,1 |

**Table S3**

|  | post op complication | | | | | | | | no post op complication | | | | | | | |
| --- | --- | --- | --- | --- | --- | --- | --- | --- | --- | --- | --- | --- | --- | --- | --- | --- |
|  | recipient bed | | | | flap | | | | recipient bed | | | | flap | | | |
|  | irradiated | | non-irradiated | | irradiated | | non-irradiated | | irradiated | | non-irradiated | | irradiated | | non-irradiated | |
| day | blood flow [AU] | | blood flow [AU] | | blood flow [AU] | | blood flow [AU] | | blood flow [AU] | | blood flow [AU] | | blood flow [AU] | | blood flow [AU] | |
|  | mean | SD | mean | SD | mean | SD | mean | SD | mean | SD | mean | SD | mean | SD | mean | SD |
| 1 | 179,5 | 50,4 | 197,6 | 78,3 | 68,1 | 34,5 | 74,9 | 48,8 | 228,1 | 58,2 | 209,2 | 60,1 | 74,6 | 40,8 | 77,0 | 44,7 |
| 2 | 215,6 | 35,4 | 236,2 | 64,9 | 90,6 | 37,3 | 87,9 | 52,5 | 231,4 | 77,6 | 221,2 | 54,6 | 78,7 | 43,3 | 84,5 | 43,5 |
| 3 | 218,6 | 65,4 | 236,6 | 60,3 | 83,2 | 41,1 | 82,3 | 53,8 | 233,2 | 72,2 | 220,4 | 49,8 | 95,3 | 54,4 | 97,2 | 49,9 |
| 5 | 174,0 | 55,7 | 250,1 | 49,6 | 101,1 | 60,8 | 105,1 | 56,0 | 230,8 | 74,3 | 229,6 | 57,1 | 103,0 | 52,7 | 105,5 | 55,4 |
| 7 | 183,1 | 59,5 | 244,1 | 57,0 | 85,5 | 36,2 | 107,4 | 55,5 | 224,3 | 62,4 | 233,4 | 53,3 | 105,7 | 50,8 | 114,3 | 55,2 |
| 14 | 187,4 | 61,6 | 235,3 | 68,5 | 102,5 | 45,6 | 135,4 | 78,5 | 213,7 | 75,8 | 229,0 | 61,3 | 108,7 | 57,5 | 115,2 | 60,7 |

**Table S4**
